# Clinical approach and utilizing liquid biopsies to interrogate suspected acquired resistance to PD-1 blockade

**DOI:** 10.2144/fsoa-2023-0010

**Published:** 2024-05-15

**Authors:** Dilanka Lakshan De Silva, Jia Luo

**Affiliations:** 1 Thoracic Oncology Service, Division of Solid Tumor Oncology, Department of Medicine, Memorial Sloan Kettering Cancer Center, New York, NY, USA; 2 Parkville Familial Cancer Centre, Peter MacCallum Centre, Melbourne, Victoria, 3000, Australia; 3 Peter MacCallum Department of Oncology, University of Melbourne Victoria, 3010, Australia; 4 Lowe Center for Thoracic Oncology, Dana-Farber Cancer Institute, Boston, MA, USA; 5 Department of Medicine, Harvard Medical School, Boston, MA, USA

**Keywords:** acquired resistance to immunotherapy, liquid biopsy, molecular sequencing, non-small cell lung cancer, PD-1 blockade, personalized medicine, precision medicine

## Abstract

PD-1 blockade is now routine for nearly all patients with non-small lung cancer. Acquired resistance to PD-1 blockade – defined generally as an initial response followed later by progression [^1-3^] is a common yet poorly understood concept. A key clinical challenge to insight has been a lack of standard guidance for clinical management of a case of suspected acquired resistance. The infrequency of performing tumor biopsies and the uncertainty of actionability from tissue sampling likely also contribute to limited insight into the biology of acquired resistance [4]. To address this knowledge gap and to highlight the value of tumor and liquid biopsy, we present a representative case of suspected acquired resistance to PD-1 blockade and propose a multi-modal guide for approaching this clinical scenario.

## Abstract (plain language summary)

Acquired resistance to immunotherapy should not be assumed but should be thoroughly interrogated.

Liquid biopsies provide invaluable real-time insights into treatment response and should be utilized whenever possible.

Incongruity in imaging, clinical status, and sequencing should not be dismissed, and an alternative explanation for the discrepancy needs to be explored.

PD-1 blockade is now routine for nearly all patients with non-small lung cancer. However, the proper investigation of alternative explanations and the cause remains not intuitive for many clinicians. We present a case of a patient who benefitted from PD-1 blockade for more than a year, but later developed what was initially suspected to be radiological progression. A systematic approach to investigating with the utilization of clinical examination, radiology and comprehensive molecular profiling with tissue and liquid biopsy of the new pulmonary nodule revealed an alternate explanation, preventing the premature discontinuation of treatment and providing a framework for approaching this clinical scenario. In presenting this individual case, we aim to highlight the approach, considerations, and tools that can be used to address new radiologic changes suspected, but not certain, to be acquired resistance to PD-1 blockade.

## Case presentation

**Figure 1.**
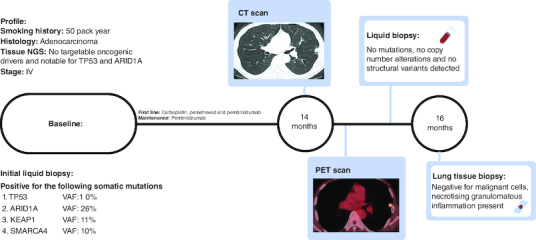
Timeline of the patient. At baseline, no targetable mutations, but liquid biopsy detected TP53, ARID1A, KEAP1 and SMARCA4 mutations. After complete response, at 14 months, a solitary pulmonary nodule was detected on CT, and it was PET avid indicative of potentially acquired resistance to PD-1 therapy. Liquid biopsy detected clearance and was inconsistent with radiology. Further investigation with tissue biopsy at 16 months revealed a benign diagnosis and refuted the progression of the disease.

A current smoker with a 50-pack-year smoking history presented to care with bilateral lung nodules and a suspicious left adrenal gland lesion. A diagnostic biopsy showed metastatic lung adenocarcinoma; scant tissue limited PD-L1 expression. Tissue underwent comprehensive molecular testing with MSK-IMPACT (Integrated Mutation Profile of Actionable Cancer Targets), the in-house FDA approved platform that can detect many mutations even at very low allele frequencies (<2%) [5]. As a companion diagnostic, he was offered MSK-ACCESS (Analysis of Circulating cfDNA to Evaluate Somatic Status), an in-house liquid biopsy platform. MSK-ACCESS can detect over 120 mutations even at a variable allele frequency of 0.5% with more than 99% sensitivity when paired with tissue sequencing, as with our patient [6]. MSK IMPACT tissue platform demonstrated no targetable mutations, but was notable for *TP53* and *ARID1A* mutations. MSK-ACCESS liquid biopsy platform was congruous with the initial tissue biopsy, but additional mutations were discovered and were notable for detectable alterations in *TP53, ARID1A, KEAP1, MAP2K* and *SMARCA4* (Figure 1). The patient initially received carboplatin, pemetrexed, and pembrolizumab and immediately achieved an excellent clinical and a complete radiological response. After four cycles of combination therapy, both carboplatin and pemetrexed were ceased and pembrolizumab was continued as maintenance therapy every 3 weeks. The deep response persisted for more than 14 months while on pembrolizumab alone, most consistent with an ongoing response to PD-1 blockade therapy.

More recently, a routine interval CT scan showed a new 1.2 cm subpleural nodule in the lingula. The prior primary and metastatic lesions remained well controlled without progression. The patient clinically felt well. To inform the persistence and avidity of the new nodule, and to assess for additional suspicious sites of distant metastasis, we evaluated his disease status with a PET scan. This test redemonstrated the PET avid new nodule with an SUVmax of 6.2 and was suspicious for recurrent malignancy but identified no other distant sites of metastasis. We also surveyed for radiologically occult systemic recurrence using ctDNA testing, which returned no detectable somatic variants, with the clearance of previously detected somatic alterations. Given the radiologic suspicion of acquired resistance to PD-1 blockade, but the patient feeling clinically well and with no detectable ctDNA shedding in the blood, we next pursued a biopsy of the nodule to determine the diagnosis. Fortunately, pathology revealed no malignant cells and rather a necrotizing, granulomatous inflammation. No further intervention was pursued on the nodule, and the patient has remained well on pembrolizumab with no further evidence of progression.

## Discussion

**Figure 2.**
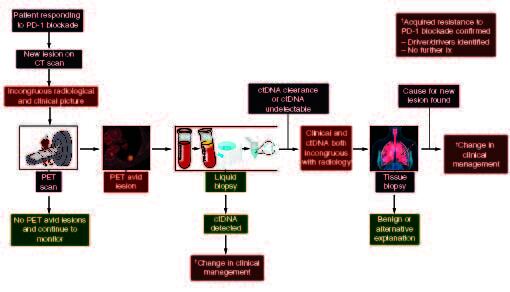
Schema for investigation of acquired resistance to PD-1 blockade. After CT diagnosis of the pulmonary nodule, proceed with functional imaging with a PET scan. If PET avid, proceed to liquid biopsy to confirm progression, identify therapeutic targets, and for consideration of management change. If the liquid biopsy confirms clearance, i.e., ongoing response, proceed to tissue biopsy to characterize the pulmonary nodule.

This case represents a common clinical scenario clinicians face in patients with non-small cell lung cancer and response to PD-1 blockade. Acquired resistance can occur in nearly 2/3rds of initial responders and, akin to this case, commonly occurs in a single site or oligo-pattern of progression [7]. Most clinicians in this context would acknowledge progression, especially after PET avidity, and explore other options such as local therapy or change of systemic treatment. Our approach to this scenario is multimodal and includes a comprehensive diagnostic evaluation rather than empirically pursuing a change in systemic therapy or palliative radiation (Figure 2). This multimodality approach utilizes comprehensive molecular profiling through tissue or liquid biopsies, traditional radiology, functional imaging and the patient's clinical status. The systematic approach helps prevent unnecessary complications associated with invasive investigations such as a tissue biopsy in a cohort of people already at high risk of complications such as pneumothorax [8,9]. This stepwise approach will guide a clinician through systemic interrogation to determine the cause for the acquired resistance and efficient deployment of resources yielding the maximum benefit to the patient and the healthcare setting.

Specifically, faced with a new nodule concerning acquired resistance to PD-1 blockade, we evaluated the patient's clinical status with an exam and history, informing the persistence relative suspicion of the nodule with a subsequent PET scan. Then we assessed the systemic status of detectable cancer with ctDNA testing to confirm the resistance and potentially identify new drivers and therapeutic options, including clinical trials if a driver was identified.

In our patient, the clinical and the complete ctDNA clearance [10] indicated ongoing response, which was incongruous with the radiology and necessitated proceeding to the final step in the scheme: a diagnostic biopsy. It is important to note that if the ctDNA was congruent with the clinical picture, it confirmed oligoprogression and could provide invaluable information regarding the underlying driver of progression and allude to targets and therapeutic options. In addition, it makes the next step, as suggested in Figure 2, proceeding to an invasive biopsy redundant. Fortunately, a benign diagnosis was discovered, and no further intervention was required preventing premature discontinuation of treatment or unnecessary radiation. This case serves as a reminder that acquired resistance should not be assumed but instead approached thoughtfully and carefully examined prior to pursuing interventions that may not be necessary or prematurely stopping the current line of therapy. Additionally, this case highlights the value of pursuing a more comprehensive molecular characterization, either from a traditional tissue biopsy or liquid biopsy, in the context of suspected acquired resistance to PD-1 blockade. This may facilitate the discovery of the molecular underpinnings of resistance and inform the development of rational therapeutic strategies in the future.

## Conclusion

A new lesion in a patient otherwise responding well to PD-1 blockade should not be presumed to be acquired resistance and should instead be investigated in a stepwise manner.
